# Long-term trends in total administered radiation dose from brain [^18^F]FDG-PET in children with drug-resistant epilepsy

**DOI:** 10.1007/s00259-024-06902-8

**Published:** 2024-10-01

**Authors:** Antonio G. Gennari, Stephan Waelti, Moritz Schwyzer, Valerie Treyer, Alexia Rossi, Thomas Sartoretti, Alexander Maurer, Georgia Ramantani, Ruth Tuura O’Gorman, Christian J. Kellenberger, Martin W. Hüllner, Michael Messerli

**Affiliations:** 1 Department of Nuclear Medicine, University Hospital Zurich, Rämistrasse 100, CH-8091 Zurich, Switzerland; 2 University of Zurich, Zurich, Switzerland; 3 Department of Radiology and Nuclear Medicine, Children’s Hospital of Eastern Switzerland, St. Gallen, Switzerland; 4 Department of Neuropediatrics, University Children’s Hospital of Zurich, Steinwiesstrasse 75, CH-8032 Zurich, Switzerland; 5 Children’s Research Centre, University Children’s Hospital of Zurich, Zurich, Switzerland; 6 MR-Research Centre, University Children’s Hospital of Zurich, Steinwiesstrasse 75, CH-8032 Zurich, Switzerland; 7 Department of Diagnostic Imaging, University Children’s Hospital of Zurich, Steinwiesstrasse 75, CH-8032 Zurich, Switzerland

**Keywords:** Pediatric, Drug-resistant epilepsy, Image quality, [^18^F]FDG-PET, Radiation dose

## Abstract

**Purpose:**

To assess the trends in administered 2-[^18^F]fluoro-2-deoxy-D-glucose ([^18^F]FDG) doses, computed tomography (CT) radiation doses, and image quality over the last 15 years in children with drug-resistant epilepsy (DRE) undergoing hybrid positron emission tomography (PET) brain scans.

**Methods:**

We retrospectively analyzed data from children with DRE who had [^18^F]FDG-PET/CT or magnetic resonance scans for presurgical evaluation between 2005 and 2021. We evaluated changes in injected [^18^F]FDG doses, administered activity per body weight, CT dose index volume (CTDIvol), and dose length product (DLP). PET image quality was assessed visually by four trained raters. Conversely, CT image quality was measured using region-of-interest analysis, normalized by signal-to-noise (SNR) and contrast-to-noise ratio (CNR).

**Results:**

We included 55 children (30 male, mean age: 9 ± 6 years) who underwent 61 [^18^F]FDG-PET scans (71% as PET/CT). Annually, the injected [^18^F]FDG dose decreased by ~ 1% (95% CI: 0.92%-0.98%, p < 0.001), with no significant changes in administered activity per body weight (p = 0.51). CTDIvol and DLP decreased annually by 16% (95% CI: 9%-23%) and 15% (95% CI: 8%-21%, both p < 0.001), respectively. PET image quality improved by 9% year-over-year (95% CI: 6%-13%, p < 0.001), while CT-associated SNR and CNR decreased annually by 7% (95% CI: 3%-11%, p = 0.001) and 6% (95% CI: 2%-10%, p = 0.008), respectively.

**Conclusion:**

Our findings indicate stability in [^18^F]FDG administered activity per body weight alongside improvements in PET image quality. Conversely, CT-associated radiation doses reduced. These results reaffirm [^18^F]FDG-PET as an increasingly safer and higher-resolution auxiliary imaging modality for children with DRE. These improvements, driven by technological advancements, may enhance the diagnostic precision and patient outcomes in pediatric epilepsy surgery.

**Supplementary Information:**

The online version contains supplementary material available at 10.1007/s00259-024-06902-8.

## Introduction

By the age of 10, up to 46 in 100,000 children are diagnosed with epilepsy [1, 2]. Approximately one-third of these children do not respond to appropriately chosen and well-tolerated anti-seizure medications and are hence classified as having drug-resistant epilepsy (DRE) [3]. A significant predictor of DRE is the presence of a structural lesion, which refers to any brain abnormality known to increase the risk of epilepsy [4]. Epilepsy surgery is a safe and effective procedure in children whose focal onset DRE arises from a non-eloquent brain region [5, 6]. The mainstays of presurgical evaluation include video-electroencephalography (EEG), magnetic resonance (MR) imaging, and neuropsychological testing [7]. Children with a solitary, discrete lesion have higher chances of achieving postsurgical seizure freedom, provided that the lesion is completely removed [5, 8]. However, over the last two decades, surgical indications have expanded to include more complex cases of extensive or even bilateral lesions and MR-negative cases [5, 9], often necessitating auxiliary tests [7].

Nuclear medicine techniques, specifically interictal positron emission tomography (PET) and ictal single-photon emission tomography (SPECT), are crucial in complex cases, confirming the lateralization or lobar localization of the presumed epileptogenic zone (EZ), which can then be targeted for resection or further investigated by invasive EEG [7]. Past studies have indicated that 2-[^18^F]fluoro-2-deoxy-D-glucose ([^18^F]FDG)-PET is instrumental in decision-making for nearly 50% of patients with incongruent findings between EEG and MR [10]. Most importantly, [^18^F]FDG-PET demonstrates lower rates of false negatives compared to SPECT [11] and helps in predicting surgical outcomes [10, 12–14].

Despite its advantages, the use of hybrid imaging techniques, particularly [^18^F]FDG-PET/computed tomography (CT), in children undergoing presurgical evaluation for DRE raises safety concerns due to the dual sources of radiation involved [15, 16]. Given the significant number of children with DRE affected by this issue, there is a notable gap in research regarding radiation trends associated with hybrid imaging techniques like [^18^F]FDG-PET/CT and [^18^F]FDG-PET/MR. Thus, this study aimed to evaluate the trends in [^18^F]FDG doses administered and the radiation doses from CT over the study period in children with DRE who underwent hybrid [^18^F]FDG-PET scans. Additionally, we assessed how these changes impacted image quality.

## Material and methods

In this retrospective, single-center study, we selected children with DRE who underwent hybrid brain [^18^F]FDG-PET scans at the Department of Nuclear Medicine, University Hospital of Zurich, for presurgical evaluation between January 1, 2005, and May 31, 2021. Inclusion criteria were: 1) DRE diagnosis, 2) hybrid [^18^F]FDG-PET brain scan, and 3) age ≤ 18 years at the time of the scan. Exclusion criteria were: 1) missing CT dose scan report, 2) congenital or acquired pathologies affecting the basal ganglia or thalamus that could interfere with image analysis (e.g., basal ganglia stroke, metabolic diseases), and 3) image artifacts that precluded evaluation.

We extracted epidemiological and epileptological data from medical records. A detailed description of these parameters is provided in the Supplementary Table 1. DRE structural etiology was classified according to Blümke et al. [17] (Table 1). For children who underwent epilepsy surgery, the structural etiology was determined based on histological analysis; those not operated were classified based on unequivocal imaging patterns on structural MR alone. Lateralization and lobar localization of the presumed EZ were determined based on seizure semiology, EEG, structural MR, PET findings, or surgical outcomes (Supplementary Table 2*)*. Lesion lateralization was subdivided into right, left, bilateral, or negative; lobar localization was classified as frontal, temporal, posterior, deep, infratentorial, or bilateral (Supplementary Table 2*)* [18–20]. In cases of unilateral lesions involving multiple lobes, the most affected lobe was identified for classification.

**Table 1:** Structural classification based on Blümke et al. [17]

	Main categories	Detailed subdivision	Cases, *n* (%)	
Structural			**42 (76%)**	
	Focal cortical dysplasia		12 (22%)	
	Hippocampal sclerosis^$^		9 (16%)	
	Low-grade glioma^$^		7 (13%)	
	MCD-other		6 (11%)	
		*Grey matter heterotopia*		*2 *
		*Tuberous sclerosis*		*2 *
		*Hypothalamic hamartoma*		*1 *
		*Polymicrogyria*		*1 *
	Non-low-grade glioma		1 (2%)	
		*High-grade glioma*		*1 *
	Vascular malformations		3 (5%)	
		*Sturge-Weber Syndrome*		*3 *
	Encephalitis		3 (5%)	
		*Rasmussen’s encephalitis*		*3 *
	Glial scarring		1 (2%)	
		*Hypoxic-ischemic encephalopathy*		*1 *
Non-structural			**13 (24%)**	
	Genetic		7 (13%)	
	Unknown*		6 (11%)	

^$^ each of these categories contain a patient scanned for persistent seizures after surgical operation; * cases in whom neither a structural etiology nor a genetic epilepsy was diagnosed; *MCD* malformation of cortical development

The local ethics committee approved the study (Number: 2020–03067). Written informed consent was waived for patients scanned before January 2016. After January 2016, only patients whose caretakers provided documented consent for the use of their medical data were included in the study.

### Image acquisition

The [^18^F]FDG-PET scans were acquired using various PET/CT and PET/MR scanners (Supplementary Table 3 and Supplementary image 1). Additional scanner information is detailed in a previous publication [21]. CT and MR were used for PET attenuation correction and anatomical co-localization of [^18^F]FDG-PET findings.

### [^18^F]FDG-PET acquisition parameters

Children were asked to fast for at least 4 to 6 h before [^18^F]FDG injection to maximize its uptake. The injected dose (MBq) was calculated according to our in-house developed protocol (Supplementary image 2). Briefly, in children weighing < 15 kg, a standard dose of 43.1 MBq was administered, which was limited by the minimum dose provided by the automatic injection system. In children with a body weight of 15–60 kg, the prepared dose was tailored to the patient’s weight and then refined using a calibration factor, with a maximal dose of 80 MBq. Finally, in children heavier than 60 kg, a standard dose of 100 MBq was administered. Minimal changes in the protocol on a per-patient basis were tolerated. If clinically indicated, children underwent sedation for image acquisition. The [^18^F]FDG uptake time was set to 50–60 min. The uptake period was spent either in the scanner (sedated children) or the waiting room (non-sedated children). In children undergoing PET/CT, CT images were acquired after PET. In children undergoing PET/MR, the MR protocol was simultaneous with PET image acquisition. The scan coverage was limited to the patient’s head. PET images were reconstructed with different reconstruction algorithms (Supplementary Table 3).

### CT acquisition parameters

The CT scan coverage was identical to the PET coverage. The CT technical parameters used are detailed in Supplementary Table 4. Standard-dose (x-ray tube voltage: 120-140 kV) or low-dose (x-ray tube voltage: 80–100 kV) protocols were adopted according to scanner generation and patient characteristics. Data on mAs and image reconstruction algorithms used throughout the study were not consistently retrievable from patients’ images or Digital Imaging and Communications in Medicine (DICOM) metadata. Therefore, they were not included in the analysis.

### MR acquisition parameters

All scans included at least 3D T1-weighted and 2D T2-weighted MR sequences of the brain. Additional sequences, such as 2D and 3D fluid-attenuated inversion recovery (FLAIR) or 3D T2-weighted sequences were acquired if needed. Sequence parameters are detailed in the Supplementary Table 5.

### Administered activity per body weight and CT dose value

The injected [^18^F]FDG dose was derived from scan reports, while administered activity per body weight was calculated by dividing the injected dose by the patient body weight (MBq/kg).

CT dose index volume (CTDIvol) and dose length product (DLP) were derived from dose reports or DICOM metadata and used to estimate the absorbed radiation dose in each patient. CTDIvol (mGy) represents the output dose of a specific scanner for a specific protocol, while DLP measures the ionizing radiation exposure during the entire acquisition (mGy*cm).

### Image quality analysis

All readings were performed on a Picture Archiving and Communication System integrated station (24-inch display, 1920 × 1080 resolution). The readers were allowed to use post-processing tools such as windowing, gradation adjustment, and magnification. Image analysis was performed as follows:

- PET scans: Four fully trained raters (two board-certified radiologists [AGG and AR] and two double board-certified radiologists and nuclear medicine physicians [AM and MWH]) qualitatively evaluated the image quality and noise of all [^18^F]FDG-PET scans using a four-point Likert scale as reported in Liberini et al. [22] (Table 2, and Supplementary image 3). Three locations were evaluated: centrum semiovale (defined as the first slice in which the corpus of the caudate nucleus is no longer visible), basal ganglia (defined as the slice in which both the head of the caudate nucleus and the putamen nuclei are visible), and cerebellum (defined as the slice in which the dentate nuclei are visible). The raters were blinded to epidemiological and epileptological information and other raters’ scores.
- CT scans: A fully trained, board-certified radiologist (AGG) drew a circular 100 mm^2^ region of interest (ROI) in the following locations: right and left thalamus, evaluated in an axial plane encompassing the genu and the splenium of the corpus callosum, as well as in the vitreous humor of the right ocular globe. The ROI’s mean density (Hounsfield units) and standard deviation (SD) were derived. Signal-to-noise ratio (SNR) and contrast-to-noise ratio (CNR) were calculated as follows:
  - SNR_CT_ = mean density values of the thalami divided by the mean value of their SDs.
  - CNR_CT_ = difference between the mean density values of the thalami and the mean density value calculated in the vitreous humor of the right ocular globe, divided by the mean value of the SDs calculated in the thalami.

**Table 2:** [^18^F]FDG-PET image quality assessment

Description		Score
Overall image quality		
Inadequate image quality with marked blurring of grey-white matter junction and cortical gyri		1
Fair image quality with diagnostically relevant blurring of grey-white matter junction and cortical gyri		2
Good image quality with diagnostically irrelevant blurring of grey-white matter junction and adequate distinction of cortical gyri		3
Very good image quality with almost any blurring of grey-white matter junction and good distinction of cortical gyri		4
Image noise		
Almost any		1
Diagnostically irrelevant		2
Diagnostically relevant		3
Marked noise		4

*[*^*18*^*F]FDG* 2-[^18^F]fluoro-2-deoxy-D-glucose*; PET* positron emission tomography

### Statistical analysis

The statistical analysis was performed using R software version *4.0.5* (https://www.r-project.org). The D’Agostino-Pearson test was used to check data distribution. Mean ± SD, median (interquartile range, IQR), frequencies, and percentages were used for descriptive statistics as appropriate. Parametric, non-parametric, Fisher’s, and Chi-square tests were used for group-wise comparisons, while Pearson and Spearman correlation tests were used to calculate the relationship between variables. Specifically, the association between epidemiological data (e.g., age at scan, weight, etc.) and the date of scan, defined as a numeric variable, were tested using Spearman’s correlation.

Quasi-Poisson regression was used to investigate the changes in injected [^18^F]FDG dose, administered activity per body weight, CTDIvol, DLP, and CT and PET image parameters from 2005 to 2021, adopting calendar year as the independent variable. Quasi-Poisson regression analysis was preferred over Poisson due to the overdispersion encountered with the latter. Age, sex, and injected [^18^F]FDG dose were included as covariates in models evaluating [^18^F]FDG-PET image quality. Only age and sex were retained in those analyzing CT dose and image quality. Age and sex were incorporated as covariates based on previous research highlighting variations in the brain according to sex and age ranges [23–25]. Models’ residuals were checked for signs of autocorrelation using the Breusch-Godfrey test. Regression coefficients were exponentially transformed and presented with their 95% confidence intervals (CIs), expressing variables’ changes over calendar years as percentages to improve the readability of the results.

To account for the foreseeable radiation dose reduction associated with a more extensive implementation of PET/MR scanners, a sensitivity analysis including both CT and MR dose values was performed. For this purpose, the CTDIvol and DLP values of MR were fictitiously set to 0.

The two-way random effect model intraclass correlation coefficient (ICC) was used to assess the inter-rater agreement on [^18^F]FDG-PET image quality and noise. Poor agreement was defined by ICC values of 0.49 and below, moderate agreement by those between 0.5 and 0.74, good agreement by those between 0.75 and 0.89, and excellent agreement by those of 0.9 and above.

All statistical analyses were two-tailed; Holm’s correction was used for multiple comparisons, with statistical significance set at *p* < 0.05.

## Results

### Patient characteristics

**Fig. 1:**
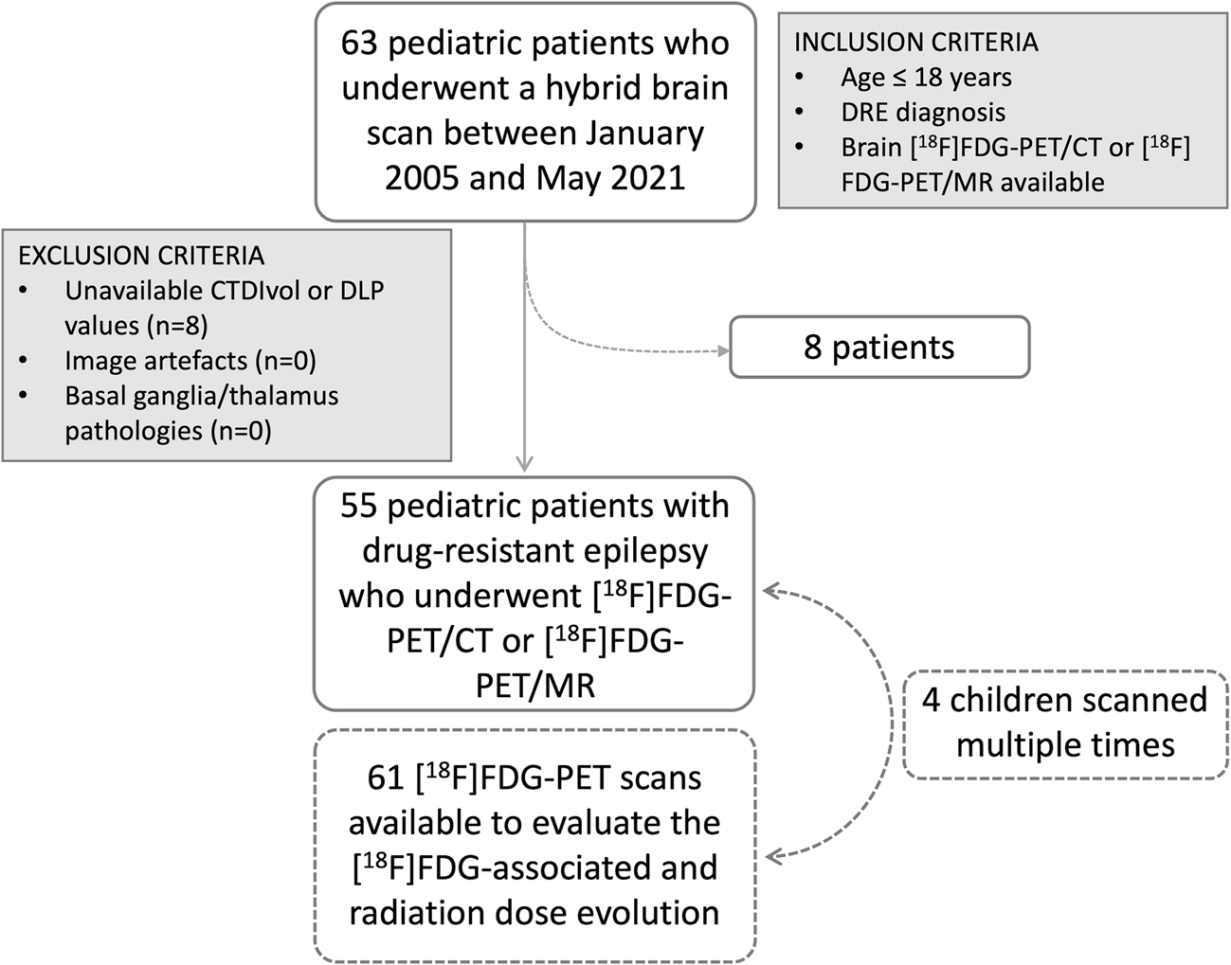
Flow diagram of the study group. CT: Computed tomography; CTDIvol: Computed tomography dose index volume; DLP: Dose-length product; DRE: Drug resistant epilepsy; [^18^F]FDG: 2-[^18^F]fluoro-2-deoxy-D-glucose; MR: Magnetic resonance; PET: Positron emission tomography

Of the 63 children initially considered for our study, eight were excluded because of unretrievable DLP and CTDIvol data (Fig. 1). Therefore, our final cohort consisted of 55 children who underwent at least one hybrid [^18^F]FDG-PET scan. The characteristics of these children and their DRE etiologies are detailed in Tables 1 and 3. Twenty-eight (51%) children had lesions detectable on structural MR, while the remaining 27 (49%) were MR-negative. Among those with detectable lesions, 36% were three years old or younger at the time of the PET scan, 7% had previously undergone epilepsy surgery for hippocampal sclerosis and low-grade epilepsy-associated tumor, respectively, and 2% presented with bilateral lesions due to tuberous sclerosis. Among MRI-negative cases, 14 (52%) were eventually diagnosed with structural DRE, whereas the rest were classified as having non-structural DRE (Table 1). Thirty-one of 55 children (53%) eventually underwent surgery, with two undergoing only biopsy and another two (4%) undergoing only depth electrode placement for invasive EEG recordings. At the time of the scan, males were older and heavier than females (*p* = 0.03 and *p* = 0.02, respectively), though there was no significant difference in BMI between sexes (*p* = 0.4). Age at scan reduced over the study period (*r* = -0.29, *p* = 0.02). Four children (three females) underwent multiple scans (Table 3), contributing to a total of 61 [^18^F]FDG-PET scans analyzed (Fig. 1); 43 (71%) of these scans were acquired using a PET/CT scanner.

**Table 3:** Patients’ characteristics

Characteristics		*n* (%)	
Patients		**55**	
Males, *n* (%)		30 (55%)	
Lesion lateralization, left, *n* (%)		23 (42%)	
Lobar localization, *n* (%)	Temporal	25 (45%)	
	Frontal	10 (18%)	
	Posterior	8 (15%)	
	Others	12 (22%)	
Detectable lesion at MR, *n* (%)		28 (51%)	
Scanned multiple times, *n* (%)		4 (7%)	
	Scanned twice	2 (50%)	• Scanned with both techniques: 1 (50%) • Scanned with PET/MR only: 1 (50%)
	Scanned three times	2 (50%)	• Scanned with both techniques: 1 (50%) • Scanned with PET/CT only: 1 (50%)
Scans evaluated		**61**	
Age at scan, y, mean ± SD		9.0 ± 5.6	
BMI, Kg/m^2^, mean ± SD		19.1 ± 5.1	
Injected [^18^F]FDG dose, MBq, median (IQR)		90 (54–100)	
Administered activity per body weight, MBq/Kg, mean ± SD		2.4 ± 1.7	
CTDIvol, mGy, median (IQR)		0.8 (0.4–2.0)	
DLP, mGy/cm, median (IQR)		14.1 (9.8–35.1)	

*BMI* Body mass index; *CTDIvol* Computed tomography dose index volume; *cm* centimeter; *DLP* Dose-length product; *[*^*18*^*F]FDG *2-[^18^F]fluoro-2-deoxy-D-glucose; *IQR* Interquartile range; *Kg* kilogram; *m* meter; *MBq* Megabecquerel; *MR* Magnetic resonance; *mGy* milligray; *SD *Standard deviation; *y* years

### [^18^F]FDG-PET activity and images

**Fig. 2:**
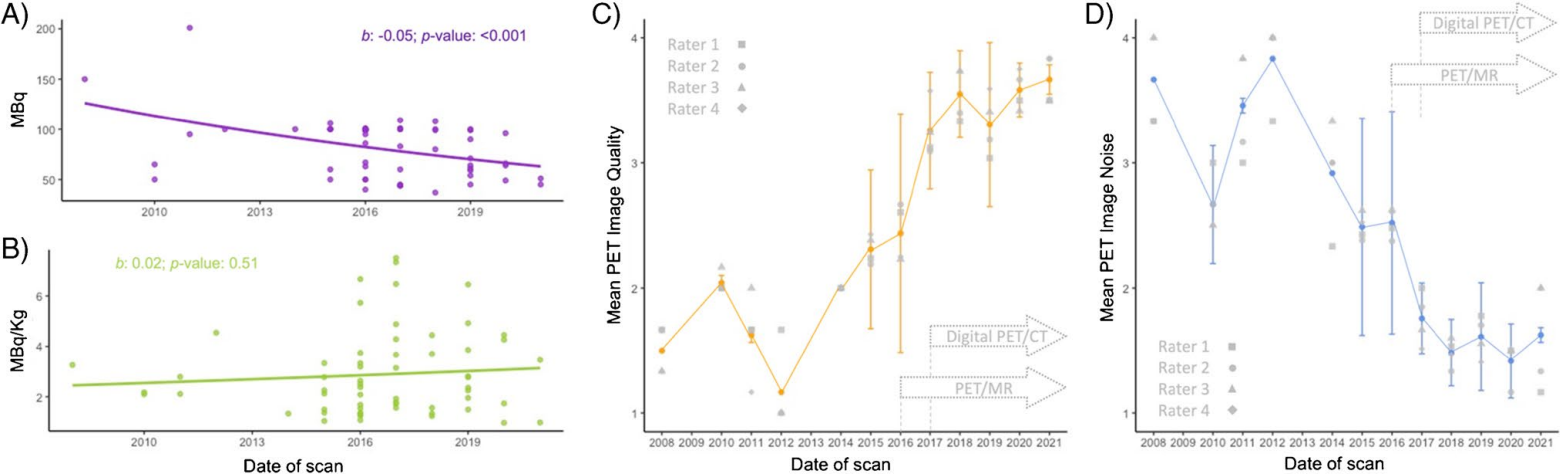
(**A, B**) Evolution of [^18^F]FDG-PET-associated parameters. Scatter plots detailing the injected (**A**) and [^18^F]FDG-administered activity per body weight (**B**) evolution over the last 15 years. Regression lines are based on quasi-Poisson regression analysis. (**C**, **D**) Estimates of the average trends of [^18^F]FDG-PET image quality and noise over the study period. Different grey symbols represent each rater’s average [^18^F]FDG-PET image quality and noise. Grey arrows detail the installation of digital PET/CT and PET/MR at our tertiary hospital. PET/MR was installed in 2014 but the first brain cases were acquired in 2016. CT: Computed tomography; [^18^F]FDG: 2-[^18^F]fluoro-2-deoxy-D-glucose; Kg: kilogram; MBq: Megabecquerel; MR: Magnetic resonance; PET: Positron emission tomography

The median injected [^18^F]FDG dose and mean administered activity per body weight are reported in Table 3. The mean administered activity per body weight was higher in females (females: 3.4 ± 1.9 MBq/kg; males: 2.4 ± 1.2 MBq/kg, *p* = 0.02). Between 2005 and 2021, a ~ 1% annual decrease occurred in the injected dose (95% CI: 0.92%-0.98%, p < 0.001), while administered activity per body weight did not vary (*p* = 0.51, Fig. 2A, B).

Twenty-one (34%) and 18 (~ 30%) [^18^F]FDG-PET scans were acquired using a digital PET/CT scanner and a PET/MR, respectively. Age at scan differed according to scanner type (p < 0.001) with median ages of 3 years (IQR: 2–7 years), 13.5 years (IQR: 12–16 years), and 12 years (IQR: 6–15 years), for PET/MR, digital PET/CT, and analogic PET/CT, respectively. In the last 15 years, [^18^F]FDG-PET image quality improved annually by 9% (95% CI: 6%-13%, p < 0.001), while noise decreased by 8% (95% CI: 6%-11%, p < 0.001, Fig. 2C, D), accounting for sex, age at scan, and injected [^18^F]FDG dose (Supplementary tables 8, 9). The combined and site-specific inter-reader agreement for [^18^F]FDG image quality and noise were excellent (Supplementary tables 6, 7)*.*

### Anatomic images

**Fig. 3:**
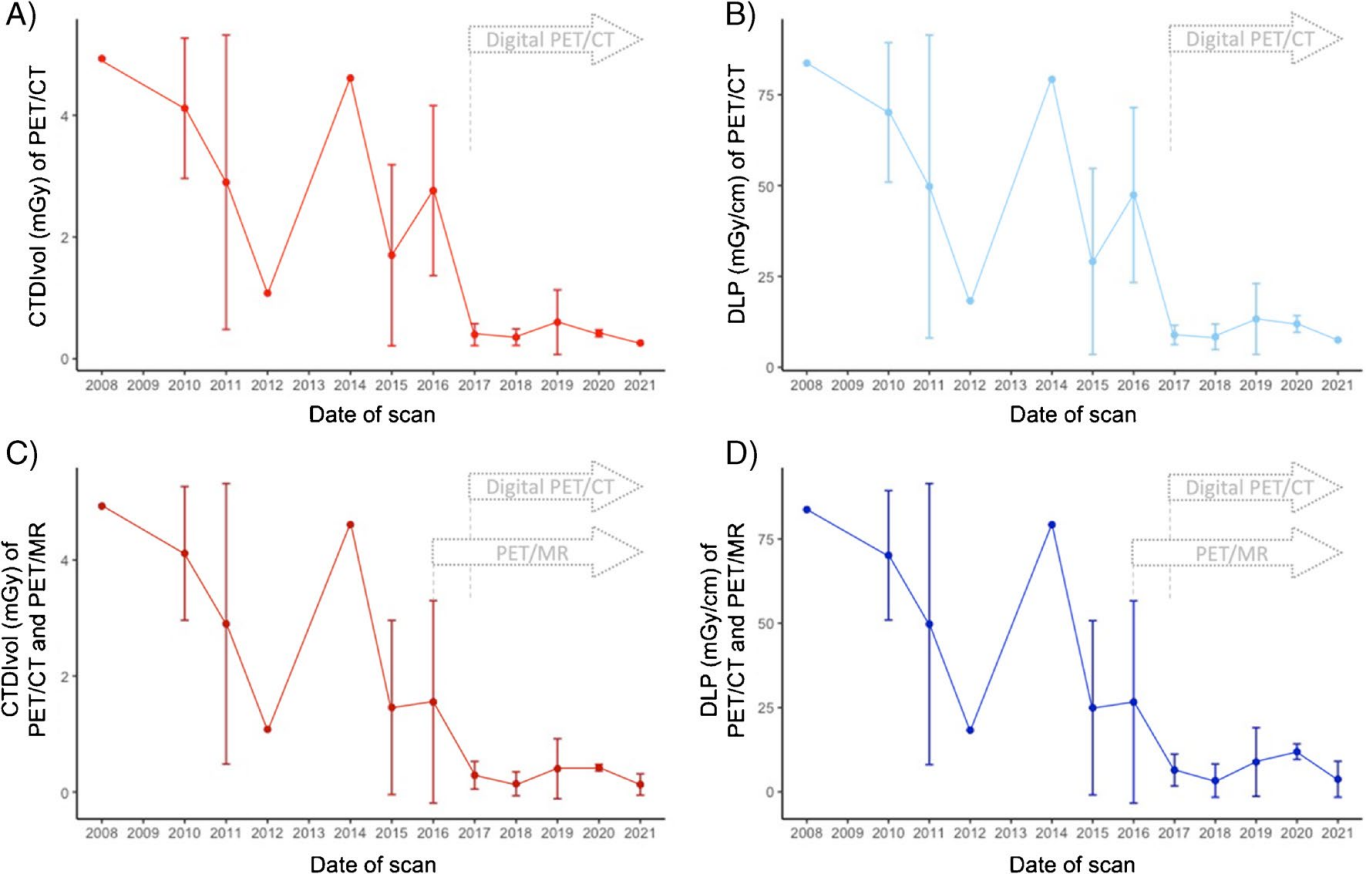
Evolution of CT-associated parameters. (**A**, **B**, **C**, **D**) Estimates of the average trends of CTDIvol and DLP over the study period. Panels (**A**) and (**B**) present the evolution of CTDIvol and DLP based on PET/CT derived data. Panels (**C**) and (**D**) present a sensitivity analysis aimed to quantify the cumulative radiation reduction in the last 15 years in children with DRE fictitiously setting PET/MR CTDIvol and DLP values to 0. Arrows detail the installation of digital PET/CT and PET/MR at our tertiary hospital. PET/MR was installed in 2014 but the first brain cases were acquired in 2016. CT: Computed tomography; CTDIvol: Computed tomography dose index volume; DLP: Dose-length product; DRE: drug resistant epilepsy; mGy: milligray; MR: Magnetic resonance; PET: Positron emission tomography

The median CTDIvol and DLP values are reported in Table 3. During the study period, the CTDIvol and DLP decreased annually by 16% (95% CI: 9%-23%) and 15% (95% CI: 8%-21%, p < 0.001 for both), respectively, accounting for sex and age at scan (Fig. 3A, B, Supplementary tables 10, 11, and Supplementary image 4). The strength of the relation describing the evolution of the radiation dose in PET/CT did not vary in the sensitivity analysis including PET/MR scans. Indeed, the CTDIvol decreased annually by 18% (95% CI: 14%-29%), while DLP by 20% (95% CI: 13%-27%, p < 0.001 for both, Fig. 3C, D, Supplementary tables 12, 13).

**Fig. 4:**
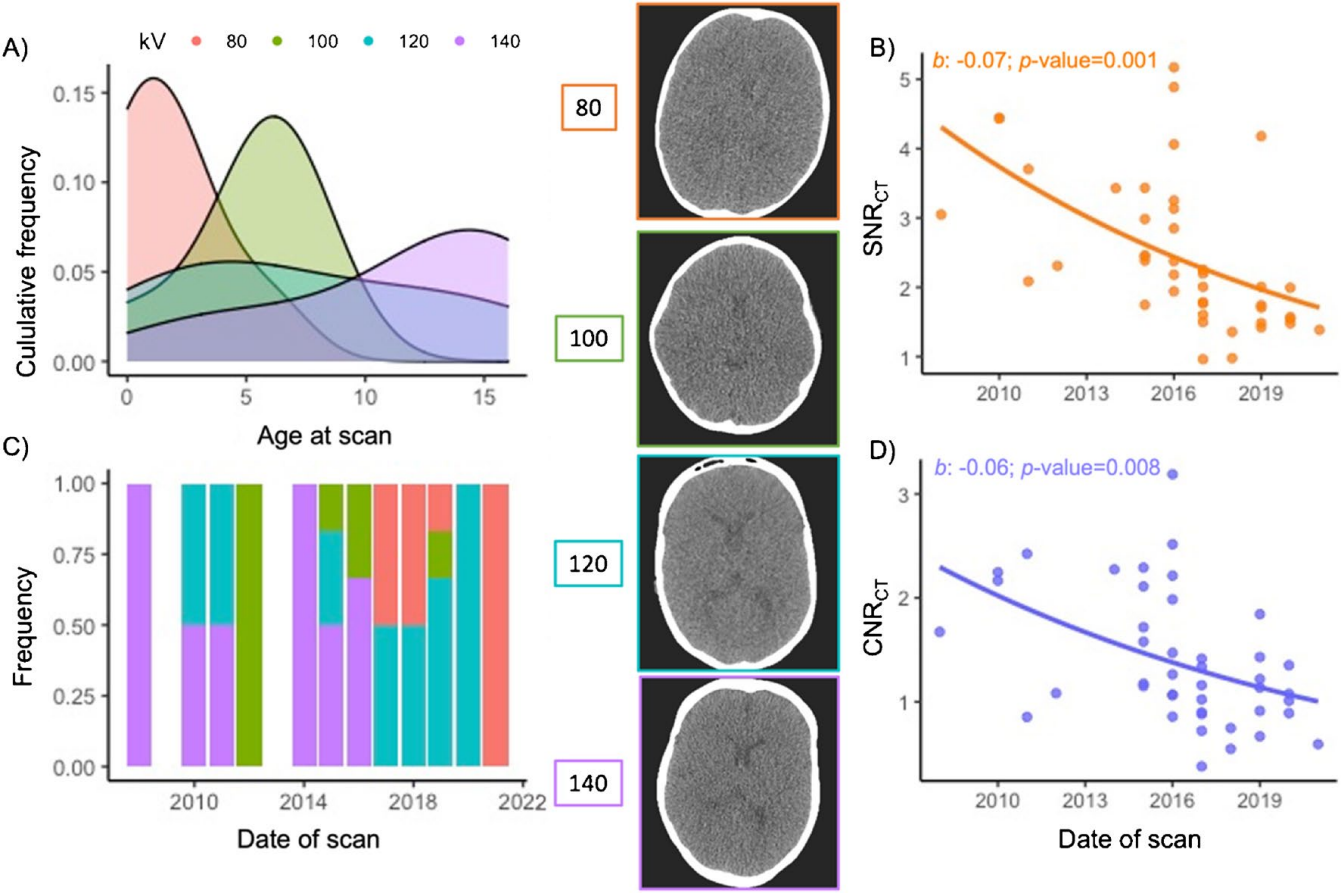
Distribution of PET/CT tube currents according to date of scan and patient age. (**A**) Density plot representing age at scan according to different kVs. Lower kVs were usually employed to scan younger children. (**C**) Percent stacked bar chart highlighting the usage of different kVs according to different years. The introduction of digital PET/CT allowed the reduction of high-dose scans. Indeed, all the exams performed at 80 kV were acquired after 2016. Exemplificative cases acquired at different kVs are provided on the side. Compared with higher kVs images, those acquired at lower kVs impeded the differentiation between grey and white matter, made ventricles recognition challenging, and had an overall more granular appearance. The ability of newer scanners to use lower kV protocols, the reduction of the age at scan throughout the study period, and the use of lower kVs protocols in younger children concurred to reduce CT image through the study period. (**B**, **D**) Scatter plots detailing the evolution of SNR and CNR over the last 15 years. Regression lines are based on quasi-Poisson regression analysis. CT: Computed tomography; CNR_CT_: Contrast-to-noise ratio; PET: Positron emission tomography; SNR_CT_: Signal-to-noise ratio; kV: kilovolt

Among the 43 PET/CT scans, 7, 6, 17, and 13 were acquired using 80, 100, 120, and 140 kV, respectively. Patient age did not significantly differ between the four groups (*p* = 0.07). Nonetheless, descriptive graphs suggested that lower kVs were commonly used in younger patients and after 2016 (Fig. 4A, C). The CTDIvol values differed at different tube voltages (*p* = 0.03), with lower CTDIvol values associated with 80 and 120 kV. Conversely, the DLP values were not affected by tube voltage (*p* = 0.06).

The SNR_CT_ and CNR_CT_ were directly related to patient age (*r* = 0.33 and *r* = 0.44; *p* = 0.039 and *p* = 0.005, respectively) and had an annualized decrease rate of 7% (95% CI: 3%-11%: p = 0.001) and 6% (95% CI: 2%-10%, p = 0.008, Fig. 4B, D, Supplementary tables 14, 15), respectively, accounting for sex and age at scan. Neither SNR_CT_ nor CNR_CT_ differed between tube voltages (*p* = 0.47 and *p* = 0.45, respectively).

## Discussion

Our study demonstrated that [^18^F]FDG-PET image quality improved annually by 9%, while CT-associated radiation dose decreased annually by 15–16%, and [^18^F]FDG administered activity per body weight remained stable over the last 15 years. Given that medical imaging is the most significant man-made source of ionizing radiation, our findings highlight the importance of the “As Low As Reasonably Achievable” principle [26, 27], particularly in children due to their long life expectancy and rapidly dividing cells [26, 27].

Compared to traditional radiological exams, [^18^F]FDG-PET/CT typically delivers higher radiation doses [28] due to its dual sources of radiation: radiopharmaceuticals and anatomical imaging [29]. Therefore, its use in children is approached with caution. Interestingly, our study detected a reduction in the injected [^18^F]FDG dose between 2005 and 2021. However, since we reported no variation in our injection protocol throughout the study period, we attribute this finding to the reduction in the age at scan, consistent with the idea of expedited epilepsy surgery in children with DRE [30]. During the study period, the European Association of Nuclear Medicine (EANM) issued several guidelines [31–34], which formed the basis for international consensus documents [35]. While the study period spans almost two decades, our [^18^F]FDG injection strategy, as well as that suggested by international guidelines and consensus documents, did not vary. This proves the generalizability of our results even if our in-house protocol differed from the EANM one. Conversely, the higher administered activity per body weight in females was explainable with random patient-based deviation from the injection protocol. Of note, the radiation concerns differ between children with oncological diseases – who require multiple scans covering broad anatomical areas, including radiosensitive structures, such as the bone marrow, thyroid, and reproductive system [36] – and those with epilepsy, who typically need a single scan. This is reflected in our findings, where 93% of patients were scanned only once. Despite the global trend of shifting from PET/CT to PET/MR, PET/CT continues to be widely used in presurgical protocols of children with DRE [10, 11, 37–39], hence, validating the ongoing tendency to reduce radiation CT-associated and/or FDG-associated doses observed in oncological scanning [21, 40] in these patients is of paramount importance.

The role of various ancillary diagnostic tools for children with DRE remains a subject of debate [7]. Different experts have favored distinct approaches based on the varying levels of confidence in these techniques. The need for an additional sedation session has been seen as a drawback, deterring pediatric neurologists and epileptologists from recommending [^18^F]FDG-PET scans for children with DRE. Nonetheless, [^18^F]FDG-PET is highly recommended for specific DRE cohorts, including those with extensive hemispheric lesions, inconclusive EEG or MR findings, or conflicting results from these modalities. These categories were well-represented in our study population, underlining the generalizability of our findings. Additionally, our results, which demonstrate substantial improvements in [^18^F]FDG-PET image quality, may bolster the credibility of this technique. Of note, literature shows that [^18^F]FDG-PET successfully localized the EZ in 72% of children deemed negative at 3T MR [41]. Further, a recent multicenter study demonstrated that short-term surgical outcomes were similar between MR-positive temporal lobe epilepsy patients with MR-EEG concordance and MR-negative patients with [^18^F]FDG-PET-EEG concordance (68% vs. 65%), underscoring the vital role of [^18^F]FDG-PET in presurgical evaluation of patients with DRE [10]. Additionally, the introduction of receptor radiotracers has proven to be more sensitive and accurate than [^18^F]FDG in localizing the EZ and is expected to yield additional improvements [36, 42].

Our study offers fresh insights into the perceived safety of hybrid scans, particularly [^18^F]FDG-PET/CT, among pediatric neurologists and epileptologists. Both standardized measures of radiation dose metrics, DLP and CTDIvol, have significantly decreased – a crucial finding for centers lacking PET/MR scanners. Notably, the CT-associated radiation doses in our study were much lower than those typically reported for traditional brain CT scans in the radiological literature [43, 44]. This reduction is increasingly relevant given the rising awareness of radiation's potential harm, making it an essential consideration for physicians and parents deciding on [^18^F]FDG-PET scans for children with DRE. In addition, ongoing technological advancements are expected to further enhance dose reduction, with newer and more efficient scanners [45].

Finally, the dissemination of PET/MR scanners could significantly reduce radiation concerns, providing a comprehensive “one-stop-shop” approach for children with DRE. The superior contrast resolution and the multiparametric capabilities of MR offer additional benefits. However, the widespread availability of such systems remains limited: currently, PET/MRs account for only ~ 1% [46] and 7% [47] of the hybrid scanners in the United States and Europe, respectively.

### Limitations

Our study's retrospective, single-center design presents certain limitations. Although conducted at one of the largest tertiary care hospitals in Switzerland, our results may not be universally applicable due to differences in healthcare systems in various countries, nor do they translate to other radiotracers. Nonetheless, our detailed technical characterization could be beneficial for other centers aiming to implement dose-reduction strategies. The lack of data on CT image acquisition (mAs) and reconstruction algorithms limited our ability to thoroughly analyze the evolution of CT image quality over time or to explore the relationship between image quality and different tube voltages (kVs). Finally, it is important to note that CTDIvol and DLP do not represent the actual or effective radiation dose absorbed by a patient, and these values can vary with different patient sizes, particularly in pediatric cases [48]. Nonetheless, these metrics are commonly used to compare scan performance across vendors and patients.

## Conclusions

Over the past fifteen years, there has been a significant decrease in the overall radiation dose associated with hybrid [^18^F]FDG-PET/CT scans in children with DRE. This trend is expected to continue with technological advancements in PET/CT scanners and the increasing availability of PET/MR scanners. These developments are likely to promote more judicious use of this advanced and valuable imaging technique.

## Supplementary Information

Below is the link to the electronic supplementary material.

Supplementary file1 (PDF 793 KB)

## Data Availability

The datasets generated during and/or analyzed during the current study are not publicly available because they are based on diagnostic images. Nonetheless, they are available from the corresponding author on reasonable request.

## References

[1] Aaberg KM, Gunnes N, Bakken IJ, Lund Soraas C, Berntsen A, Magnus P, et al. Incidence and Prevalence of Childhood Epilepsy: A Nationwide Cohort Study. Pediatrics. 2017;139. 10.1542/peds.2016-3908. 10.1542/peds.2016-3908 28557750

[2] Camfield CS, Camfield PR, Gordon K, Wirrell E, Dooley JM. Incidence of epilepsy in childhood and adolescence: a population-based study in Nova Scotia from 1977 to 1985. Epilepsia. 1996;37:19–23. 10.1111/j.1528-1157.1996.tb00506.x. 8603618 10.1111/j.1528-1157.1996.tb00506.x

[3] Kwan P, Arzimanoglou A, Berg AT, Brodie MJ, Allen Hauser W, Mathern G, et al. Definition of drug resistant epilepsy: consensus proposal by the ad hoc Task Force of the ILAE Commission on Therapeutic Strategies. Epilepsia. 2010;51:1069–77. 10.1111/j.1528-1167.2009.02397.x. 19889013 10.1111/j.1528-1167.2009.02397.x

[4] Kwan P, Schachter SC, Brodie MJ. Drug-resistant epilepsy. N Engl J Med. 2011;365:919–26. 10.1056/NEJMra1004418. 21899452 10.1056/NEJMra1004418

[5] Cross JH, Reilly C, Gutierrez Delicado E, Smith ML, Malmgren K. Epilepsy surgery for children and adolescents: evidence-based but underused. Lancet Child Adolesc Health. 2022;6:484–94. 10.1016/S2352-4642(22)00098-0. 35568054 10.1016/S2352-4642(22)00098-0

[6] Ramantani G. Epilepsy surgery in early life: the earlier, the better. World Neurosurgery. 2019;131:285–6. 10.1016/j.wneu.2019.08.084. 31658555 10.1016/j.wneu.2019.08.084

[7] Jayakar P, Gaillard WD, Tripathi M, Libenson MH, Mathern GW, Cross JH, et al. Diagnostic test utilization in evaluation for resective epilepsy surgery in children. Epilepsia. 2014;55:507–18. 10.1111/epi.12544. 24512473 10.1111/epi.12544

[8] Pindrik J, Hoang N, Smith L, Halverson M, Wojnaroski M, McNally K, et al. Preoperative evaluation and surgical management of infants and toddlers with drug-resistant epilepsy. Neurosurg Focus. 2018;45:E3. 10.3171/2018.7.FOCUS18220. 30173613 10.3171/2018.7.FOCUS18220

[9] Eriksson MH, Whitaker KJ, Booth J, Piper RJ, Chari A, Martin Sanfilippo P, et al. Pediatric epilepsy surgery from 2000 to 2018: Changes in referral and surgical volumes, patient characteristics, genetic testing, and postsurgical outcomes. Epilepsia. 2023;64:2260–73. 10.1111/epi.17670. 37264783 10.1111/epi.17670 PMC7615891

[10] Steinbrenner M, Duncan JS, Dickson J, Rathore C, Wachter B, Aygun N, et al. Utility of 18F-fluorodeoxyglucose positron emission tomography in presurgical evaluation of patients with epilepsy: A multicenter study. Epilepsia. 2022;63:1238–52. 10.1111/epi.17194. 35166379 10.1111/epi.17194

[11] Lascano AM, Perneger T, Vulliemoz S, Spinelli L, Garibotto V, Korff CM, et al. Yield of MRI, high-density electric source imaging (HD-ESI), SPECT and PET in epilepsy surgery candidates. Clin Neurophysiol. 2016;127:150–5. 10.1016/j.clinph.2015.03.025. 26021550 10.1016/j.clinph.2015.03.025

[12] Sarikaya I. PET studies in epilepsy. Am J Nucl Med Mol Imaging. 2015;5:416–30. 26550535 PMC4620171

[13] Fernandez S, Donaire A, Seres E, Setoain X, Bargallo N, Falcon C, et al. PET/MRI and PET/MRI/SISCOM coregistration in the presurgical evaluation of refractory focal epilepsy. Epilepsy Res. 2015;111:1–9. 10.1016/j.eplepsyres.2014.12.011. 25769367 10.1016/j.eplepsyres.2014.12.011

[14] Toth M, Barsi P, Toth Z, Borbely K, Luckl J, Emri M, et al. The role of hybrid FDG-PET/MRI on decision-making in presurgical evaluation of drug-resistant epilepsy. BMC Neurol. 2021;21:363. 10.1186/s12883-021-02352-z. 34537017 10.1186/s12883-021-02352-z PMC8449490

[15] Yuan MK, Chang SC, Yuan MC, Foo NP, Chan SH, Wang SY, et al. Pediatric nuclear medicine examinations and subsequent risk of neoplasm: a nationwide population-based cohort study. Front Med (Lausanne). 2021;8:764849. 10.3389/fmed.2021.764849. 34988089 10.3389/fmed.2021.764849 PMC8720959

[16] Earl VJ, Baker LJ, Perdomo AA. Effective doses and associated age-related risks for common paediatric diagnostic nuclear medicine and PET procedures at a large Australian paediatric hospital. J Med Imaging Radiat Oncol. 2022;66:7–13. 10.1111/1754-9485.13257. 34110081 10.1111/1754-9485.13257

[17] Blumcke I, Spreafico R, Haaker G, Coras R, Kobow K, Bien CG, et al. Histopathological findings in brain tissue obtained during epilepsy surgery. N Engl J Med. 2017;377:1648–56. 10.1056/NEJMoa1703784. 29069555 10.1056/NEJMoa1703784

[18] Gennari AG, Cserpan D, Stefanos-Yakoub I, Kottke R, O’Gorman Tuura R, Ramantani G. Diffusion tensor imaging discriminates focal cortical dysplasia from normal brain parenchyma and differentiates between focal cortical dysplasia types. Insights Imaging. 2023;14:36. 10.1186/s13244-023-01368-y. 36826756 10.1186/s13244-023-01368-y PMC9958211

[19] Gennari AG, Bicciato G, Lo Biundo SP, Kottke R, Stefanos-Yakoub I, Cserpan D, et al. Lesion volume and spike frequency on EEG impact perfusion values in focal cortical dysplasia: a pediatric arterial spin labeling study. Sci Rep. 2024;14:7601. 10.1038/s41598-024-58352-9. 38556543 10.1038/s41598-024-58352-9 PMC10982306

[20] Stefanos-Yakoub I, Wingeier K, Cserpan D, Gennari AG, Latal B, Reuner G, et al. Lesion extent negatively impacts intellectual skills in pediatric focal epilepsy. Pediatr Neurol. 2023;145:67–73. 10.1016/j.pediatrneurol.2023.05.005. 37285765 10.1016/j.pediatrneurol.2023.05.005

[21] Waelti S, Skawran S, Sartoretti T, Schwyzer M, Gennari AG, Mader C, et al. A third of the radiotracer dose: two decades of progress in pediatric [(18)F]fluorodeoxyglucose PET/CT and PET/MR imaging. Eur Radiol. 2023. 10.1007/s00330-023-10319-6. 37855853 10.1007/s00330-023-10319-6 PMC11126459

[22] Liberini V, Pizzuto DA, Messerli M, Orita E, Grunig H, Maurer A, et al. BSREM for brain metastasis detection with 18F-FDG-PET/CT in lung cancer patients. J Digit Imaging. 2022;35:581–93. 10.1007/s10278-021-00570-y. 35212859 10.1007/s10278-021-00570-y PMC9156589

[23] Murphy DG, DeCarli C, McIntosh AR, Daly E, Mentis MJ, Pietrini P, et al. Sex differences in human brain morphometry and metabolism: an in vivo quantitative magnetic resonance imaging and positron emission tomography study on the effect of aging. Arch Gen Psychiatry. 1996;53:585–94. 10.1001/archpsyc.1996.01830070031007. 8660125 10.1001/archpsyc.1996.01830070031007

[24] Brickman AM, Buchsbaum MS, Shihabuddin L, Hazlett EA, Borod JC, Mohs RC. Striatal size, glucose metabolic rate, and verbal learning in normal aging. Brain Res Cogn Brain Res. 2003;17:106–16. 10.1016/s0926-6410(03)00085-5. 12763197 10.1016/s0926-6410(03)00085-5

[25] Chugani HT. A critical period of brain development: studies of cerebral glucose utilization with PET. Prev Med. 1998;27:184–8. 10.1006/pmed.1998.0274. 9578992 10.1006/pmed.1998.0274

[26] Kutanzi KR, Lumen A, Koturbash I, Miousse IR. Pediatric exposures to ionizing radiation: carcinogenic considerations. Int J Environ Res Public Health. 2016;13. 10.3390/ijerph13111057. 10.3390/ijerph13111057 PMC5129267 27801855

[27] Andreassi MG, Picano E. Reduction of radiation to children: our responsibility to change. Circulation. 2014;130:135–7. 10.1161/CIRCULATIONAHA.114.010699. 24914036 10.1161/CIRCULATIONAHA.114.010699

[28] Mettler FA Jr, Mahesh M, Bhargavan-Chatfield M, Chambers CE, Elee JG, Frush DP, et al. Patient exposure from radiologic and nuclear medicine procedures in the United States: procedure volume and effective dose for the Period 2006–2016. Radiology. 2020;295:418–27. 10.1148/radiol.2020192256. 32181730 10.1148/radiol.2020192256 PMC9754695

[29] Kaste SC. PET-CT in children: where is it appropriate? Pediatr Radiol. 2011;41(Suppl 2):509–13. 10.1007/s00247-011-2096-1. 21847731 10.1007/s00247-011-2096-1 PMC4698349

[30] Hale AT, Chari A, Scott RC, Helen Cross J, Rozzelle CJ, Blount JP, et al. Expedited epilepsy surgery prior to drug resistance in children: a frontier worth crossing? Brain. 2022;145:3755–62. 10.1093/brain/awac275. 35883201 10.1093/brain/awac275

[31] Lassmann M, Biassoni L, Monsieurs M, Franzius C, Jacobs F, Dosimetry E, et al. The new EANM paediatric dosage card. Eur J Nucl Med Mol Imaging. 2007;34:796–8. 10.1007/s00259-007-0370-0. 17406866 10.1007/s00259-007-0370-0

[32] Lassmann M, Biassoni L, Monsieurs M, Franzius C, Dosimetry E, Paediatrics C. The new EANM paediatric dosage card: additional notes with respect to F-18. Eur J Nucl Med Mol Imaging. 2008;35:1666–8. 10.1007/s00259-008-0799-9. 18574583 10.1007/s00259-008-0799-9

[33] Lassmann M, Treves ST, Group ESPDHW. Paediatric radiopharmaceutical administration: harmonization of the 2007 EANM paediatric dosage card (version 1.5.2008) and the 2010 North American consensus guidelines. Eur J Nucl Med Mol Imaging. 2014;41:1036–41. 10.1007/s00259-014-2731-9. 24599377 10.1007/s00259-014-2731-9

[34] Guedj E, Varrone A, Boellaard R, Albert NL, Barthel H, van Berckel B, et al. EANM procedure guidelines for brain PET imaging using [(18)F]FDG, version 3. Eur J Nucl Med Mol Imaging. 2022;49:632–51. 10.1007/s00259-021-05603-w. 34882261 10.1007/s00259-021-05603-w PMC8803744

[35] Tian M, Watanabe Y, Kang KW, Murakami K, Chiti A, Carrio I, et al. International consensus on the use of [(18)F]-FDG PET/CT in pediatric patients affected by epilepsy. Eur J Nucl Med Mol Imaging. 2021;48:3827–34. 10.1007/s00259-021-05524-8. 34453559 10.1007/s00259-021-05524-8

[36] Madar I, Lesser RP, Krauss G, Zubieta JK, Lever JR, Kinter CM, et al. Imaging of delta- and mu-opioid receptors in temporal lobe epilepsy by positron emission tomography. Ann Neurol. 1997;41:358–67. 10.1002/ana.410410311. 9066357 10.1002/ana.410410311

[37] Dangouloff-Ros V, Fillon L, Eisermann M, Losito E, Boisgontier J, Charpy S, et al. Preoperative detection of subtle focal cortical dysplasia in children by combined arterial spin labeling, voxel-based morphometry, electroencephalography-synchronized functional MRI, resting-state regional homogeneity, and 18F-fluorodeoxyglucose positron emission tomography. Neurosurgery. 2022. 10.1227/neu.0000000000002310. 36700754 10.1227/neu.0000000000002310

[38] Boscolo Galazzo I, Mattoli MV, Pizzini FB, De Vita E, Barnes A, Duncan JS, et al. Cerebral metabolism and perfusion in MR-negative individuals with refractory focal epilepsy assessed by simultaneous acquisition of (18)F-FDG PET and arterial spin labeling. Neuroimage Clin. 2016;11:648–57. 10.1016/j.nicl.2016.04.005. 27222796 10.1016/j.nicl.2016.04.005 PMC4872676

[39] Ding Y, Zhu Y, Jiang B, Zhou Y, Jin B, Hou H, et al. (18)F-FDG PET and high-resolution MRI co-registration for pre-surgical evaluation of patients with conventional MRI-negative refractory extra-temporal lobe epilepsy. Eur J Nucl Med Mol Imaging. 2018;45:1567–72. 10.1007/s00259-018-4017-0. 29671038 10.1007/s00259-018-4017-0

[40] Skawran S, Sartoretti T, Gennari AG, Schwyzer M, Sartoretti E, Treyer V, et al. Evolution of CT radiation dose in pediatric patients undergoing hybrid 2-[(18)F]FDG PET/CT between 2007 and 2021. Br J Radiol. 2023;96:20220482. 10.1259/bjr.20220482. 37751216 10.1259/bjr.20220482 PMC10646648

[41] Kim YH, Kang HC, Kim DS, Kim SH, Shim KW, Kim HD, et al. Neuroimaging in identifying focal cortical dysplasia and prognostic factors in pediatric and adolescent epilepsy surgery. Epilepsia. 2011;52:722–7. 10.1111/j.1528-1167.2010.02950.x. 21275980 10.1111/j.1528-1167.2010.02950.x

[42] Muzik O, da Silva EA, Juhasz C, Chugani DC, Shah J, Nagy F, et al. Intracranial EEG versus flumazenil and glucose PET in children with extratemporal lobe epilepsy. Neurology. 2000;54:171–9. 10.1212/wnl.54.1.171. 10636144 10.1212/wnl.54.1.171

[43] Brady Z, Ramanauskas F, Cain TM, Johnston PN. Assessment of paediatric CT dose indicators for the purpose of optimisation. Br J Radiol. 2012;85:1488–98. 10.1259/bjr/28015185. 22844033 10.1259/bjr/28015185 PMC3500792

[44] Eddy FK, Ngano SO, Jerve FA, Serge A. Radiation dose evaluation of pediatric patients in CT brain examination: multi-center study. Sci Rep. 2021;11:4663. 10.1038/s41598-021-84078-z. 33633210 10.1038/s41598-021-84078-z PMC7907073

[45] Tschauner S, Zellner M, Pistorius S, Gnannt R, Schraner T, Kellenberger CJ. Ultra-low-dose lung multidetector computed tomography in children – Approaching 0.2 millisievert. Eur J Radiol. 2021;139. 10.1016/j.ejrad.2021.109699. 10.1016/j.ejrad.2021.109699 33932715

[46] Ehman EC, Johnson GB, Villanueva-Meyer JE, Cha S, Leynes AP, Larson PEZ, et al. PET/MRI: Where might it replace PET/CT? J Magn Reson Imaging. 2017;46:1247–62. 10.1002/jmri.25711. 28370695 10.1002/jmri.25711 PMC5623147

[47] European Society for Hybrid MaTI. MAP of PET/MR systems in Europe per city.

[48] Strauss KJ. Radiation safety summit – method to estimate radiation dose to pediatric patients from CT scans. Pediatr Radiol. 2011;41:210–1. 10.1007/s00247-011-1978-6.

