# Supplementary material for: Long-term trends in total administered radiation dose from brain [18F]FDG-PET in children with drug-resistant epilepsy
Source: Eur J Nucl Med Mol Imaging. 2024 Oct 1;52(2):574–85. doi: 10.1007/s00259-024-06902-8 (PMC11732939; doi:10.1007/s00259-024-06902-8)
Supplement: Supplementary file 1 — Supplementary file1 (PDF 793 KB) [file 259_2024_6902_MOESM1_ESM.pdf]

Supplementary Material

SUPPLEMENTARY TABLE 1 – DETAILED DESCRIPTION OF EPIDEMIOLOGICAL AND EPILEPTOLOGICAL PARAMETERS RETRIEVED FROM MEDICAL RECORDS. 2

SUPPLEMENTARY TABLE 2 – DETAILED DESCRIPTION OF LESION LATERALIZATION AND LOBAR LOCALIZATION. 2

SUPPLEMENTARY TABLE 3 – SCANNERS AND IMAGE RECONSTRUCTION ALGORITHMS USED. 3

SUPPLEMENTARY TABLE 4 – CT ACQUISITION PARAMETERS. 3

SUPPLEMENTARY TABLE 5 – MR ACQUISITION PARAMETERS. 4

SUPPLEMENTARY TABLE 6 –ASSESSMENT OF INTER-RATER IMAGE QUALITY AGREEMENT. 5

SUPPLEMENTARY TABLE 7 –ASSESSMENT OF INTER-RATER IMAGE NOISE AGREEMENT. 6

SUPPLEMENTARY TABLE 8 – STATISTICAL MODEL PRESENTING [^{18}F]FDG-PET IMAGE QUALITY EVOLUTION. 7

SUPPLEMENTARY TABLE 9 – STATISTICAL MODEL PRESENTING [^{18}F]FDG-PET IMAGE NOISE EVOLUTION. 7

SUPPLEMENTARY TABLE 10 – STATISTICAL MODEL PRESENTING CTDI EVOLUTION FOCUSING UNIQUELY ON PET-CT. 8

SUPPLEMENTARY TABLE 11 – STATISTICAL MODEL PRESENTING DLP EVOLUTION FOCUSING UNIQUELY ON PET-CT. 8

SUPPLEMENTARY TABLE 12 – STATISTICAL MODEL PRESENTING CTDI EVOLUTION INCLUDING PET-MR. 9

SUPPLEMENTARY TABLE 13 – STATISTICAL MODEL PRESENTING DLP EVOLUTION INCLUDING PET-MR. 9

SUPPLEMENTARY TABLE 14 – STATISTICAL MODEL CT SIGNAL-TO-NOISE RATIO EVOLUTION. 10

SUPPLEMENTARY TABLE 15 – STATISTICAL MODEL CT CONTRAST-TO-NOISE RATIO EVOLUTION. 10

SUPPLEMENTARY IMAGE 1 – EXEMPLIFICATIVE CASES ACCORDING TO DIFFERENT SCANNERS. 11

SUPPLEMENTARY IMAGE 2 – [^{18}F]FDG INJECTION PROTOCOL DIFFERENCES BETWEEN INTERNATIONAL GUIDELINES (1) AND OUR IN-HOUSE PROTOCOL. 12

SUPPLEMENTARY IMAGE 3 – EXEMPLIFICATIVE CASES WITH DIFFERENT [^{18}F]FDG PET IMAGE QUALITY. 13

SUPPLEMENTARY IMAGE 4 – CTDIVOL (EXPRESSED IN MGY) AND DLP (EXPRESSED IN MGY-CM) USE ACCORDING TO SCAN DATE. 14

BIBLIOGRAPHY 15

Supplementary table 1 – Detailed description of epidemiological and epileptological parameters retrieved from medical records.

	Parameters retrieved
**Epidemiological**	
	Sex
	Age at scan
	Height
	Weight
	Body mass index
	Visibility of a lesion on previous MR scans
	PET findings
	Operation status and history of previous brain surgery
**Epileptological**	
	Lateralization of the presumed epileptogenic region*
	Lobar localization of the presumed epileptogenic region*

**: the lateralization and lobar location of the presumed epileptogenic region were established based on semiology, EEG, structural MR, PET findings, and surgical results (in children who underwent surgery); EEG: electroencephalography; MR: magnetic resonance; PET: positron emission tomography*

Supplementary table 2 – Detailed description of lesion lateralization and lobar localization.

	Parameters retrieved
**Lesion lateralization**	
	Right
	Left
	Bilateral
	Negative
**Lobar localization**	
	Frontal
	Temporal
	Posterior (lesions located in the parietal or occipital lobe or combination of both)
	Deep (lesions located in the insula, basal ganglia, thalamus, or hypothalamus)
	Infratentorial (cerebellar)
	Bilateral (lesion involving both hemispheres simultaneously, i.e., tuberous sclerosis)

Supplementary table 3 – Scanners and image reconstruction algorithms used.

	Type	Name	Scans, n(%)	
**Scanner**				
	**PET/CT**		43 (70%)	
		*Discovery RX**		*10 (16%)*
		*Discovery HR**		*1 (2%)*
		*Discovery 690**		*3 (5%)*
		*Discovery 710**		*1 (2%)*
		*Discovery STE**		*9 (14%)*
		*Discovery MI**		*19 (31%)*
	**PET/MR**		18 (30%)	
		*SIGNA**		*18 (30%)*
**Image reconstruction algorithm**				
		QCFX	22 (36%)	
		VPFXS	19 (31%)	
		FORE FBP 3D	20 (33%)	

: all scanners were from the same manufacturer, GE Healthcare, Waukesha, WI, USA. CT: Computed tomography; MR: Magnetic resonance; PET: Positron emission tomography

Supplementary table 4 – CT acquisition parameters.

	Details
Acquisition	Spiral
Collimation	0.625 mm
Slice thickness	3,75 mm
Reconstruction kernel	Standard or soft

CT: Computed tomography, mm: millimeter

Supplementary table 5 – MR acquisition parameters.

	TE (msec)	TR (msec)	TI (msec)	Flip Angle	Averages	Slice thickness (mm)	Spacing between slices (mm)
3D T1 FSPGR	0.02-3	8-930		5-12	1	1-1.2	0.5-0.6
2D T2 weighted	13	614-916		20/25	1	3-4	3.3-5.3
3D T2 weighted	111-121	11000		111	2	3	3
2D FLAIR*	88-92	8000	2356	160	1	3-4	3.3-5.3
3D FLAIR*	137.97	7002	1865	90	1	1.2	0.6

**=FLAIR sequence was acquired optionally; FLAIR = fluid attenuated inversion recovery; FSPGR = fast spoiled gradient echo; mm = millimeter; MR: Magnetic resonance; msec = milliseconds; TE = echo time; TI = inversion time; TR = repetition time*

Supplementary table 6 –Assessment of inter-rater image quality agreement.

	Rater 1	Rater 2	Rater 3	Combined inter-rater agreement
**Mean quality**				0.95 (0.92 to 0.96)
Rater 2*	0.97 (0.94 to 0.98)			
Rater 3*	0.84 (0.73 to 0.90)	0.87 (0.78 to 0.92)		
Rater 4*	0.84 (0.74 to 0.91)	0.88 (0.79 to 0.83)	0.97 (0.95 to 0.98)	
**Centrum semiovale**				0.94 (0.91 to 0.96)
Rater 2*	0.93 (0.89 to 0.96)			
Rater 3*	0.85 (0.75 to 0.91)	0.86 (0.77 to 0.92)		
Rater 4*	0.83 (0.72 to 0.90)	0.85 (0.75 to 0.91)	0.96 (0.93 to 0.97)	
**Basal Ganglia**				0.93 (0.89 to 0.95)
Rater 2*	0.91 (0.85 to 0.95)			
Rater 3*	0.81 (0.68 to 0.88)	0.83 (0.72 to 0.90)		
Rater 4*	0.80 (0.67 to 0.88)	0.82 (0.71 to 0.89)	0.96 (0.94 to 0.98)	
**Cerebellum**				0.90 (0.85 to 0.94)
Rater 2*	0.92 (0.84 to 0.95)			
Rater 3*	0.71 (0.52 to 0.83)	0.70 (0.50 to 0.82)		
Rater 4*	0.78 (0.62 to 0.87)	0.82 (0.70 to 0.89)	0.93 (0.88 to 0.96)	

**: Measures are presented as intraclass correlation coefficient (confidence interval)*

Supplementary table 7 –Assessment of inter-rater image noise agreement.

	Rater 1	Rater 2	Rater 3	Combined inter-rater agreement
**Mean noise**				0.95 (0.92 to 0.97)
Rater 2*	0.96 (0.93 to 0.97)			
Rater 3*	0.83 (0.72 to 0.90)	0.88 (0.80 to 0.93)		
Rater 4*	0.85 (0.76 to 0.91)	0.90 (0.83 to 0.94)	0.98 (0.97 to 0.99)	
**Centrum semiovale**				0.92 (0.89 to 0.95)
Rater 2*	0.88 (0.80 to 0.93)			
Rater 3*	0.77 (0.62 to 0.86)	0.85 (0.76 to 0.91)		
Rater 4*	0.77 (0.62 to 0.86)	0.87 (0.78 to 0.92)	0.97 (0.95 to 0.98)	
**Basal Ganglia**				0.93 (0.98 to 0.95)
Rater 2*	0.93 (0.89 to 0.95)			
Rater 3*	0.79 (0.65 to 0.87)	0.82 (0.70 to 0.89)		
Rater 4*	0.81 (0.69 to 0.89)	0.82 (0.71 to 0.89)	0.98 (0.97 to 0.99)	
**Cerebellum**				0.94 (0.91 to 0.96)
Rater 2*	0.94 (0.89 to 0.96)			
Rater 3*	0.82 (0.70 to 0.89)	0.85 (0.74 to 0.91)		
Rater 4*	0.85 (0.75 to 0.91)	0.86 (0.77 to 0.92)	0.95 (0.92 to 0.97)	

**: Measures are presented as intraclass correlation coefficient (confidence interval)*

Supplementary table 8 – Statistical model presenting [^{18}F]FDG-PET image quality evolution.

	Estimate	Rate Ratio	Rate Ratio Confidence Interval	p-value
**Year of Scan**	9.1×10^{-2}	1.09	1.06 to 1.13	1.1×10^{-7}
**Age at Scan**	6.1×10^{-3}	1.01	0.99 to 1.02	0.50
**Sex (Male)**	-2.7×10^{-3}	1.00	0.88 to 1.14	0.97
**MBq injected**	8.5×10^{-4}	1.00	1.00 to 1.00	0.66

[^{18}F]FDG: 2-[^{18}F]fluoro-2-deoxy-D-glucose; MBq: Mega Becquerel; PET: Positron emission tomography

Supplementary table 9 – Statistical model presenting [^{18}F]FDG-PET image noise evolution.

	Estimate	Rate Ratio	Rate Ratio Confidence Interval	p-value
**Year of Scan**	$-8.8 \text{ x } 10^{-2}$	0.92	0.89 to 0.94	5.6×10^{-7}
**Age at Scan**	$-6.5 \text{ x } 10^{-3}$	0.99	0.97 to 1.01	0.53
**Sex (Male)**	$-1.7 \text{ x } 10^{-2}$	0.98	0.83 to 1.16	0.84
**MBq injected**	$-1.1 \text{ x } 10^{-4}$	1.00	1.00 to 1.00	0.95

[^{18}F]FDG: 2-[^{18}F]fluoro-2-deoxy-D-glucose; MBq: Mega Becquerel; PET: Positron emission tomography

Supplementary table 10 – Statistical model presenting CTDIvol evolution focusing uniquely on PET-CT.

	Estimate	Rate Ratio	Rate Ratio Confidence Interval	p-value
**Year of Scan**	-0.18	0.84	0.77 to 0.91	9.9×10^{-5}
**Age at Scan**	0.04	1.05	1.0 to 1.1	0.048
**Sex (Male)**	-0.38	0.68	0.39 to 1.2	0.63

CT: Computed tomography; CTDIvol: Computed tomography dose index volume; PET: Positron emission tomography

Supplementary table 11 – Statistical model presenting DLP evolution focusing uniquely on PET-CT.

	Estimate	Rate Ratio	Rate Ratio Confidence Interval	p-value
**Year of Scan**	-0.15	0.85	0.79 to 0.92	1.8×10^{-4}
**Age at Scan**	0.04	1.05	1.00 to 1.09	0.047
**Sex (Male)**	-0.31	0.73	0.44 to 1.22	0.24

CT: Computed tomography; DLP: Dose length product; PET: Positron emission tomography

Supplementary table 12 – Statistical model presenting CTDIvol evolution including PET-MR.

	Estimate	Rate Ratio	Confidence interval of the rate ratio	p-value
**Year of Scan**	-0.24	0.78	0.71 to 0.86	4.7×10^{-6}
**Age at Scan**	-1×10^{-3}	1	0.94 to 1.06	0.96
**Sex (Male)**	-0.17	0.84	0.42 to 1.67	0.63

CTDIvol: Computed tomography dose index volume; PET: Positron emission tomography; MR: Magnetic resonance

Supplementary table 13 – Statistical model presenting DLP evolution including PET-MR.

	Estimate	Rate Ratio	Confidence interval of the rate ratio	p-value
**Year of Scan**	-0.22	0. 80	0.73 to 0.87	4.7×10^{-6}
**Age at Scan**	-6.9×10^{-3}	0.99	0.94 to 1.05	0.96
**Sex (Male)**	-0.10	0.90	0.47 to 1.72	0.63

DLP: Dose length product; PET: Positron emission tomography; MRI: Magnetic resonance

Supplementary table 14 – Statistical model CT signal-to-noise ratio evolution.

	Estimate	Rate Ratio	Confidence interval of the rate ratio	p-value
**Year of Scan**	-0.07	0. 93	0.89 to 0.97	0.001
**Age at Scan**	9.3×10^{-3}	1.01	0.99 to 1.03	0.43
**Sex (Male)**	-0.17	0.84	0.65 to 1.09	0.20

CT: Computed tomography

Supplementary table 15 – Statistical model CT contrast-to-noise ratio evolution.

	Estimate	Rate Ratio	Confidence interval of the rate ratio	p-value
**Year of Scan**	-0.06	0. 94	0.90 to 0.98	0.008
**Age at Scan**	0.02	1.02	1.00 to 1.05	0.09
**Sex (Male)**	-0.21	0.81	0.62 to 1.06	0.13

CT: Computed tomography

Supplementary image 1 – Exemplificative cases according to different scanners.

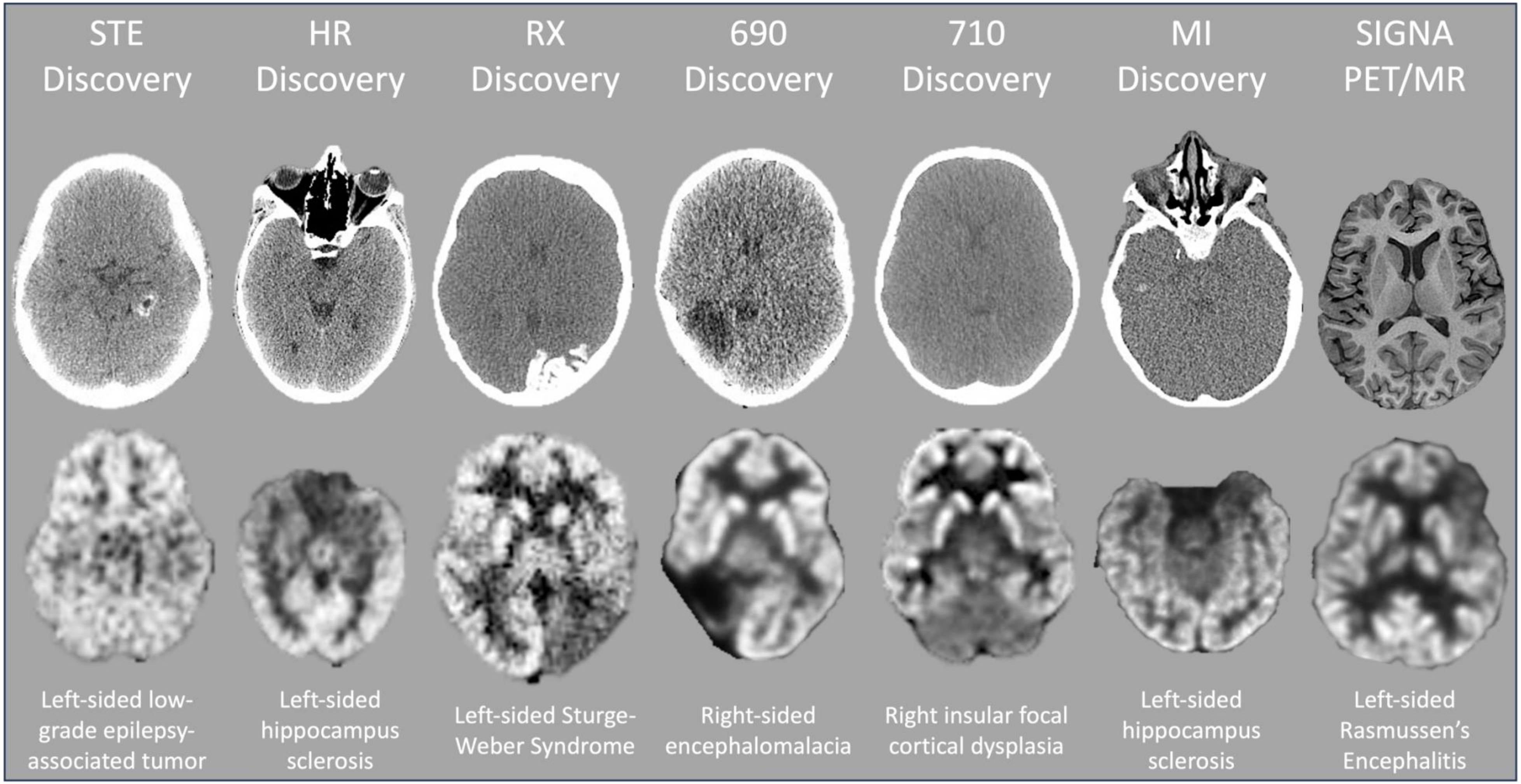

Seven different cases illustrate different pathologies, CT, [^{18}F]FDG-PET, and MRI findings. All pathologies associated with hypometabolic areas on [^{18}F]FDG-PET images. Additionally, the top and bottom rows highlight CT and [^{18}F]FDG-PET image quality changes according to different scanners. On CT images, brain cisterns and lateral ventricles were more easily definable with older scanners (i.e. STE Discovery and HR Discovery) than with newer ones (i.e., MI Discovery) due to the lower kVs protocols used with newer scanners. Conversely, the recognizability of brain gyri on [^{18}F]FDG-PET images drastically improved with the introduction of newer scanners. CT: Computed tomography; [^{18}F]FDG: 2-[18F]fluoro-2-deoxy-D-glucose; PET: Positron emission tomography; MR: Magnetic resonance

Supplementary image 2 – [^{18}F]FDG injection protocol differences between international guidelines (1) and our in-house protocol.

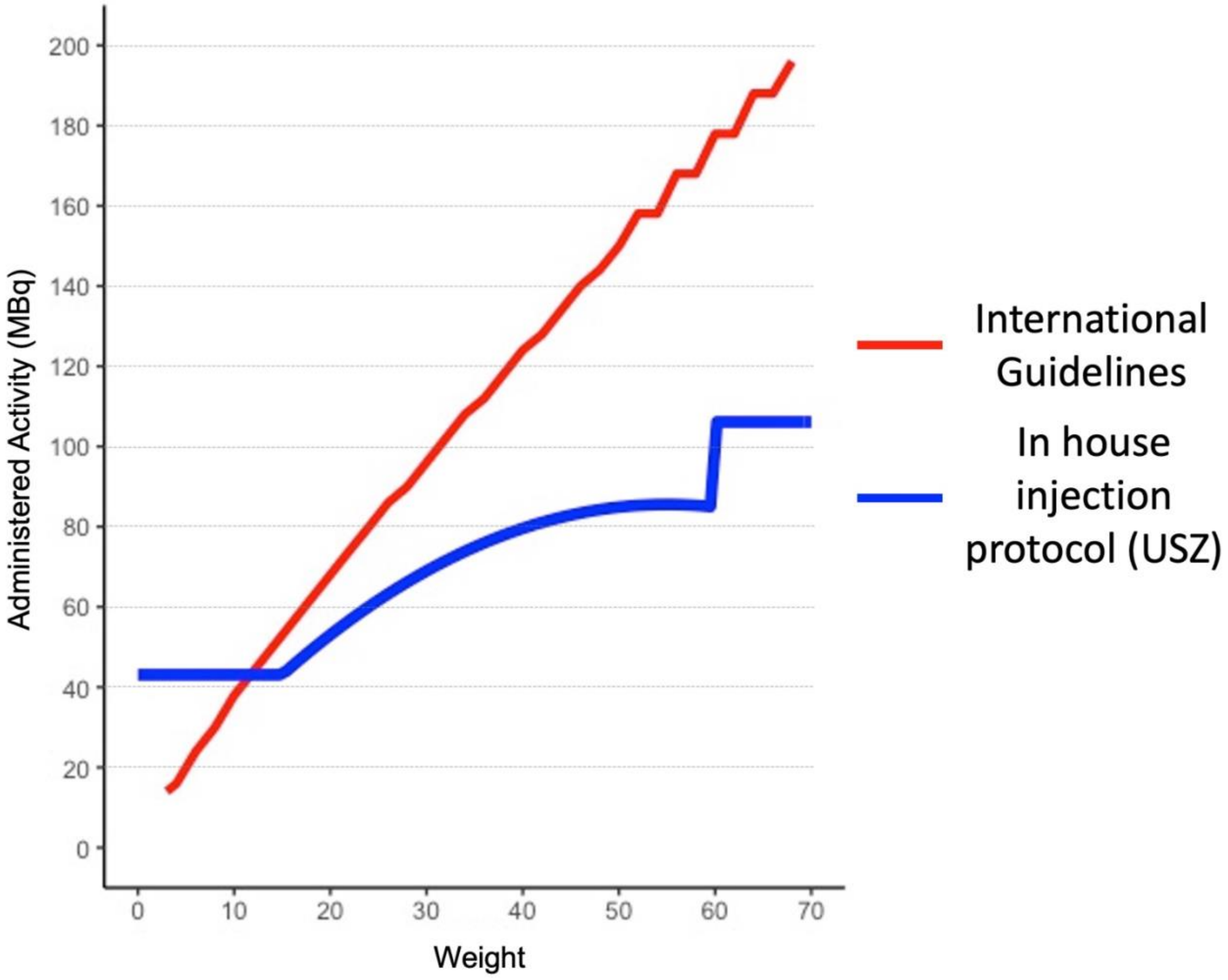

[^{18}F]FDG: 2-[^{18}F]fluoro-2-deoxy-D-glucose; MBq: Mega Becquerel; USZ: University Hospital Zurich

Supplementary image 3 – Exemplificative cases with different [^{18}F]FDG-PET image quality.

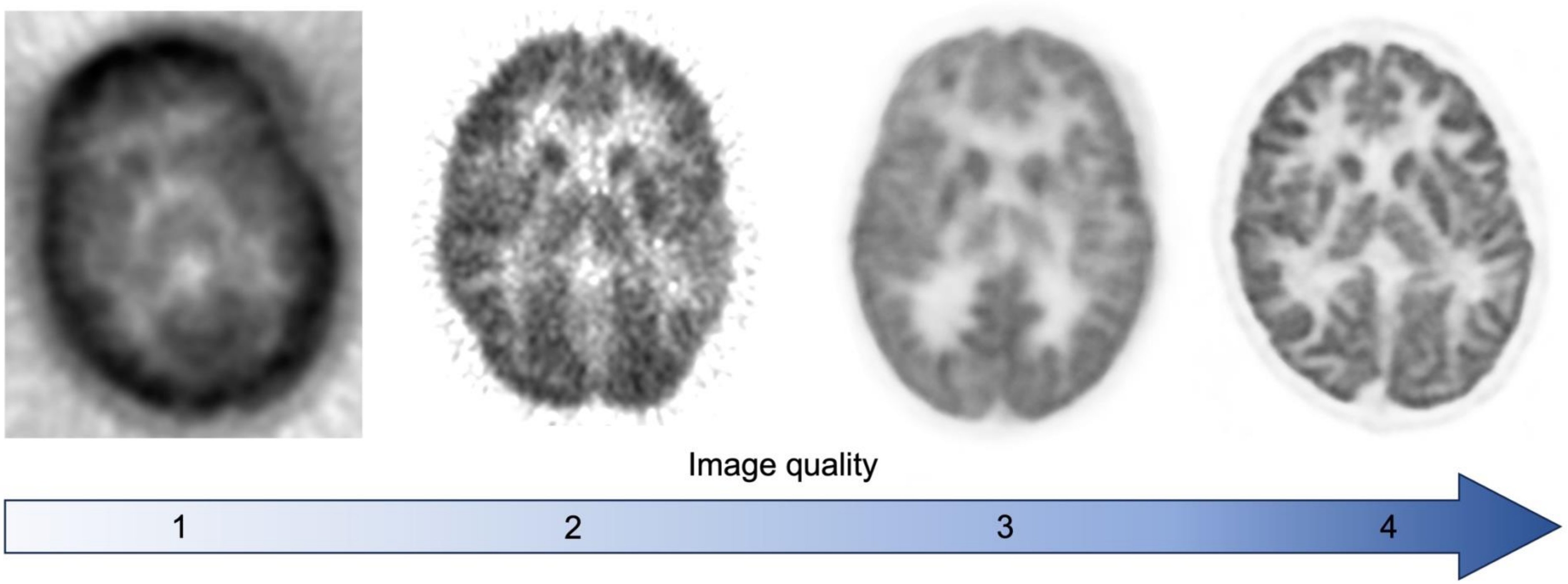

Four different cases illustrate various levels of image quality. The lowest level (marked as 1) shows a cortical gyral architecture that is scarcely recognizable alongside an ill-defined grey-to-white matter junction. Conversely, in the highest level (marked as 4), the sulci and the gyri are clearly delineated, and the grey-to-white matter junction is well-defined, enabling precise separation between the two tissues. [^{18}F]FDG: 2-[18F]fluoro-2-deoxy-D-glucose; PET: Positron emission tomography

Supplementary image 4 – CTDIvol (expressed in mGy) and DLP (expressed in mGy-cm) use according to scan date.

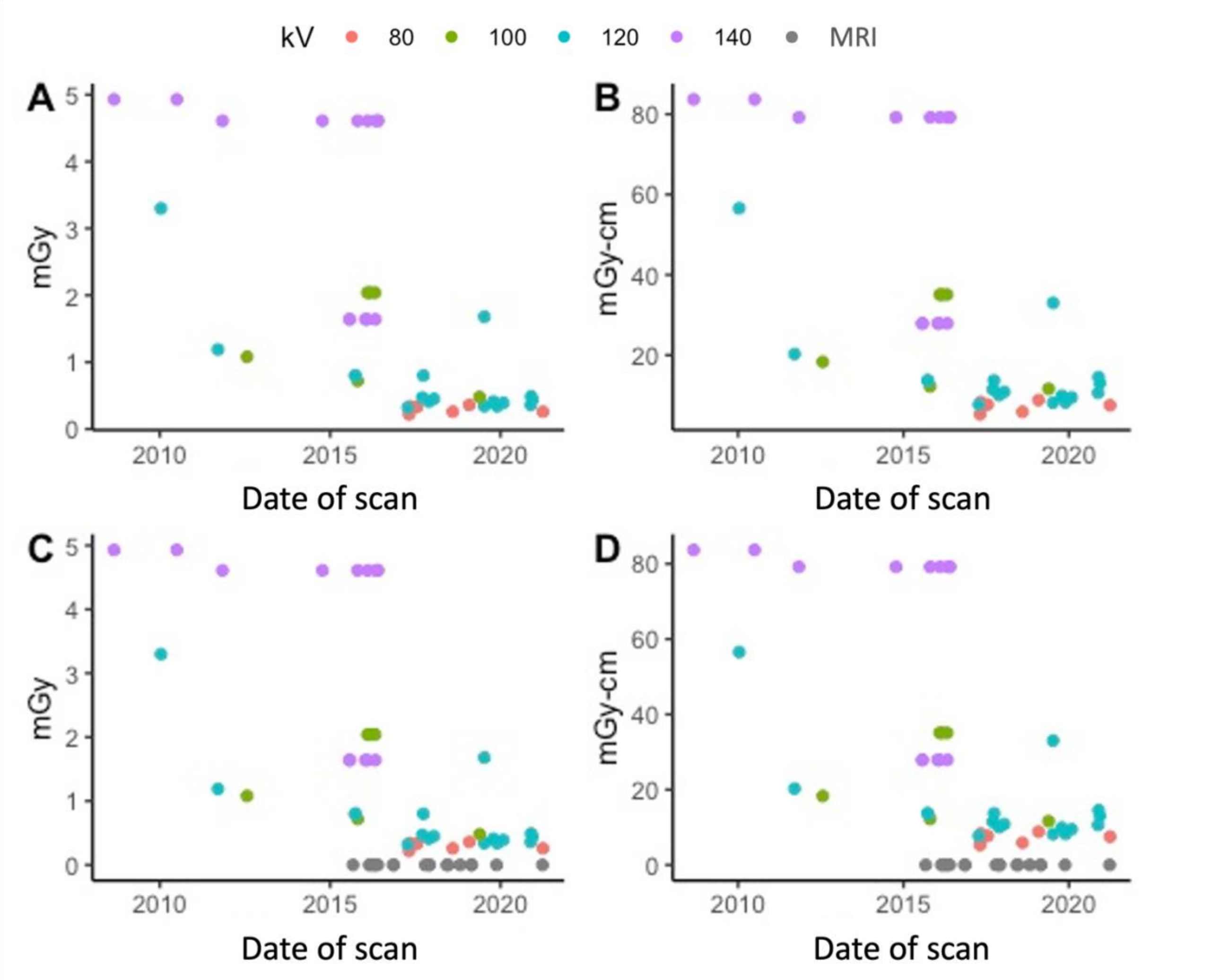

Scatter plots providing CTDIvol (A, C) and DLP (B, D) values according to date, colored according to different tube voltages. The data presented in this figure are similar to those of Figure 3, differing in data representation. While Figure 3 presents scans aggregating them according to the year of scan, here scan date is treated as a continuous variable. Scatterplots A and B show CT-associated dose information, while C and D presents data derived by a sensitivity analysis fictitiously setting PET/MR CTDIvol and DLP to 0. cm: centimeters, CT: computed tomography, CTDIvol: computed tomography dose index volume, DLP: dose-length product, PET: positron emission tomography, MR: magnetic resonance, mGy: milligray.

Bibliography

1. Tian M, Watanabe Y, Kang KW, Murakami K, Chiti A, Carrio I, et al. International consensus on the use of [(18)F]-FDG PET/CT in pediatric patients affected by epilepsy. Eur J Nucl Med Mol Imaging. 2021;48(12):3827-34.
